# Determination of the Cytosolic NADPH/NADP Ratio in *Saccharomyces cerevisiae* using Shikimate Dehydrogenase as Sensor Reaction

**DOI:** 10.1038/srep12846

**Published:** 2015-08-05

**Authors:** Jinrui Zhang, Angela ten Pierick, Harmen M. van Rossum, Reza Maleki Seifar, Cor Ras, Jean-Marc Daran, Joseph J. Heijnen, S. Aljoscha Wahl

**Affiliations:** 1 Department of Biotechnology, Delft University of Technology, Delft, 2628BC, The Netherlands

## Abstract

Eukaryotic metabolism is organised in complex networks of enzyme catalysed reactions which are distributed over different organelles. To quantify the compartmentalised reactions, quantitative measurements of relevant physiological variables in different compartments are needed, especially of cofactors. NADP(H) are critical components in cellular redox metabolism. Currently, available metabolite measurement methods allow whole cell measurements. Here a metabolite sensor based on a fast equilibrium reaction is introduced to monitor the cytosolic NADPH/NADP ratio in *Saccharomyces cerevisiae*: 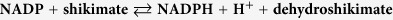. The cytosolic NADPH/NADP ratio was determined by measuring the shikimate and dehydroshikimate concentrations (by GC-MS/MS). The cytosolic NADPH/NADP ratio was determined under batch and chemostat (aerobic, glucose-limited, D = 0.1 h^−1^) conditions, to be 22.0 ± 2.6 and 15.6 ± 0.6, respectively. These ratios were much higher than the whole cell NADPH/NADP ratio (1.05 ± 0.08). In response to a glucose pulse, the cytosolic NADPH/NADP ratio first increased very rapidly and restored the steady state ratio after 3 minutes. In contrast to this dynamic observation, the whole cell NADPH/NADP ratio remained nearly constant. The novel cytosol NADPH/NADP measurements provide new insights into the thermodynamic driving forces for NADP(H)-dependent reactions, like amino acid synthesis, product pathways like fatty acid production or the mevalonate pathway.

The ratios of NAD/NADH and NADPH/NADP (nicotinamide adenine dinucleotide (phosphate)) determine the intracellular redox potential which influence the thermodynamic driving force of many reactions *in vivo*. However, the cofactors are compartmentalised in *Saccharomyces cerevisiae*, especially NADP(H) itself is not transported between compartments in *S. cerevisiae*. Furthermore, the majority of NAD(P)H is found to be protein- or enzyme-bound *in vivo* in erythrocytes (in rat) and plant systems^1^,^2^, but only the fraction of free cofactors actually drives the reaction rates^3^. Currently, most measurement techniques only allow to measure whole cell amounts, which cannot distinguish between different compartments nor between free and protein-bound concentrations. These whole cell measurements are not sufficient for a comprehensive understanding and description of the *in vivo* thermodynamics of compartmentalised reactions, like for amino acid synthesis and product pathways like farnesene.

In *S. cerevisiae,* the NADPH producing reactions, e.g. glucose-6-P dehydrogenase (G6PDH) and 6-phosphogluconate dehydrogenase (6PGDH) of the Pentose Phosphate Pathway (PPP), are reactions with large thermodynamic driving force, which allows to achieve a high NADPH/NADP ratio. In contrast NADPH consuming reactions typically operate close to the equilibrium^4^, therefore the NADPH/NADP concentration ratio has a significant contribution to the driving force of the synthesis reactions, e.g. fatty acids, amino acids and products from the mevalonic acid pathway.

The fatty acids are important precursors for the production of fuels and chemicals^5^. Fatty acids biosynthesis is initiated by the reduction of acetyl-CoA and CO_2_ to malonyl-CoA (in the cytosol), with NADPH as cofactor. A high cytosolic NADPH/NADP ratio (high driving force) is critical to obtain high production rates. A high cytosolic NADPH level is also a critical bottleneck in amino acid production, one of the largest classes of fermentation products whose syntheses are closely correlated with the availability of NADPH, e.g. L-arginine^6^ and L-lysine^7^. Additionally, there are many other reduced product reactions which depend on a high reduction potential. One prominent example is reducing pathways with aldehyde intermediates, which are toxic, and the concentration in the cell needs to be kept low. The aldehyde is usually reduced to alcohols (-OH) by NADH- or NADPH- dependent dehydrogenases. One example is the microbial production of 1,3-propanediol (PDO) in *Escherichia coli*. It was found that by switching the NADH-dependent dehydrogenase DhaT to the NADPH-dependent dehydrogenase YqhD for the conversion of the toxic aldehyde 3-HPA to PDO, a higher PDO titer was achieved and the differences in the cofactor reduced/oxidised ratios contributed to the high PDO titer^8^,^9^.

Subcellular quantification has been approached by non-aqueous fractionation in mammalian tissues and plants, a successful approach but very laborious^10^. Transcriptional sensor was used to monitor the intracellular NADH/NAD redox state^11^. Alternatively, metabolite sensor reactions have been proposed to monitor compartment specific concentrations (or ratios), especially for cofactor couples like NAD/NADH, NADPH/NADP, and ATP/ADP^12^,^13^,^14^,^15^. Malic enzyme and isocitrate dehydrogenase have been used to determine the cytosolic free NADPH/NADP ratio in rat liver and mouse pancreatic islets^13^,^16^. Especially, the cytosolic free NADPH/NADP ratio was found to correlate with increasing extracellular glucose concentrations. Ratios up to 57.8 were found which was 1 to 2 orders of magnitude different from the whole-cell NADPH/NADP ratio (approx. 1.2)^16^. Unfortunately, both reactions (malic enzyme and isocitrate dehydrogenase) and all previously described sensor reactions can’t be applied as sensor reaction in *S. cerevisiae* because of their localization in the cytosol and/or mitochondria, and reported unspecific cofactor binding (NAD or NADP)^17^,^18^.

Here we focus on the cytosolic free NADPH/NADP ratio by using a heterologous metabolite sensor reaction. For the proper function as a sensor reaction, several criteria have to be fulfilled^15^,^19^:(1) the enzyme is specific for its coenzyme; (2) the enzyme activity has to be high enough to establish equilibrium between the reactants; (3) the equilibrium constant (K_eq_) has to be known; (4) the reaction and the measured reactants (except the ones to be determined) are only present in the compartment of interest and can be measured. The reaction can be a dead-end or within a pathway, as long as the enzyme capacity is sufficient to reach pseudo equilibrium.

A heterologous candidate reaction is shikimate dehydrogenase (EC 1.1.1.25), which is NADP specific and only in the cytosol in *E. coli*^20^,^21^. The specific activity of shikimate dehydrogenase (a multifunctional arom enzyme) in *S. cerevisiae* was very low^22^,^23^, which is also only in the cytosol^24^. Therefore, the shikimate dehydrogenase from *E. coli*, which has a high activity (19.9 units/mg)^25^ was overexpressed in yeast CEN.PK 113-5D, and we assume the expressed shikimate dehydrogenase activity is only cytosolic and the SA/DHS accumulates only in the cytosol. With this reaction in place, the cytosolic NADPH/NADP ratio under different batch, chemostat and dynamic perturbation conditions was studied.

## Results and Discussion

### NADPH/NADP Metabolite sensor reaction

The genetically introduced NADPH/NADP sensor reaction is catalysed by shikimate dehydrogenase (EC 1.1.1.25) (*aroE*):


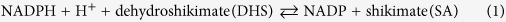



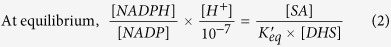


Both SA and DHS can be measured using metabolomics approach (GS-MS/MS, Supplementary figure S1). The apparent equilibrium constant K_eq_’ is 0.26 at specified reference conditions of pH = 7.0, and ionic strength I = 0.25 M^26^. The reported NADPH/NADP ratios here were calculated assuming intracellular pH = 7.0.

In the cellular system NADPH is a conserved moiety. In case of a reaction a proton is released, while a proton is produced when NADPH is oxidised.


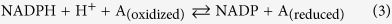



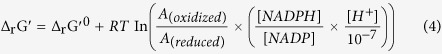



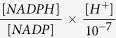
 can be replaced by the measured SA/DHS ratio, the Gibbs energy of a reduction reaction became:


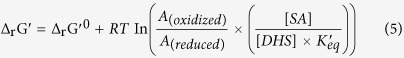


Please note that this substitution shows that for the thermodynamic analysis of any reaction involving NADPH/NADP, no assumption on the pH is required (cancels out). For the ratio itself an assumption is needed, but this does not bias the thermodynamic interpretations. Consequently, even if the pH is estimated incorrect, the Δ_r_G′ difference of the NADP(H) independent reaction is calculated correctly. Clearly, this also holds true under dynamic conditions, like the cytosolic pH would rapidly drop after the glucose pulse^27^,^28^,^29^,^30^,^31^, and the effect of changes of the cytosolic pH on the calculated thermodynamic driving force are circumvented. This is another advantage to use the sensor cytosolic NADPH/NADP ratio instead of whole cell ratio measurements.

### Influence of the sensor reaction on cell physiology

The successful expression of shikimate dehydrogenase was confirmed by enzymatic activity measurement of cell extracts. The CEN.PK-aroE strain displayed a high shikimate dehydrogenase activity (9.68 U/mg_protein_, batch condition), while the reference strain only reached 0.048 U/mg_protein_. The activity of G6PDH was comparable in both the CEN.PK-aroE and reference strain (0.70 vs. 0.57 U/mg_protein_).

In *S. cerevisiae*, the oxidative Pentose Phosphate Pathway (oxPPP) is the major source of NADPH in the cytoplasm, and it has been observed that 2% to 60% of the consumed glucose is oxidised in the oxPPP under aerobic conditions^32^,^33^. Assuming the maximum rate of oxPPP is 60% of the maximum glucose uptake rate (2.9 μmol/(gDW∙s)^34^, a NADPH production rate of 1.74 μmol/(gDW∙s) is obtained. Assuming 1 gDW contains 330 mg soluble protein^35^, the SDH activity of 9.68 U/mg protein (at 25 °C) corresponds to a maximal activity of 53.24 μmol/(gDW∙s), about 15 times higher (maybe even higher at 30 °C) than the estimated maximal NADPH flux, suggesting that the SA dehydrogenase enzyme activity is high enough to keep the sensor reaction close to equilibrium.

To analyse whether the sensor reaction influences the metabolism, the batch growth rate and metabolite levels during steady state were compared to wildtype values. The batch growth rate of CEN.PK-aroE was 0.32 h^−1^, similar to the reference strain (CEN.PK113-7D) rate (0.33 h^−1^). There was no significant difference between intracellular concentrations of most intermediates in central metabolism of the two strains under glucose-limited steady state conditions (D = 0.1 h^−1^) (Supplementary Table S1). This indicates that the expression of shikimate dehydrogenase had no considerable impact on the central metabolism under these growth conditions.

### External supplementation of SA for the sensor reaction

**Figure 1:**
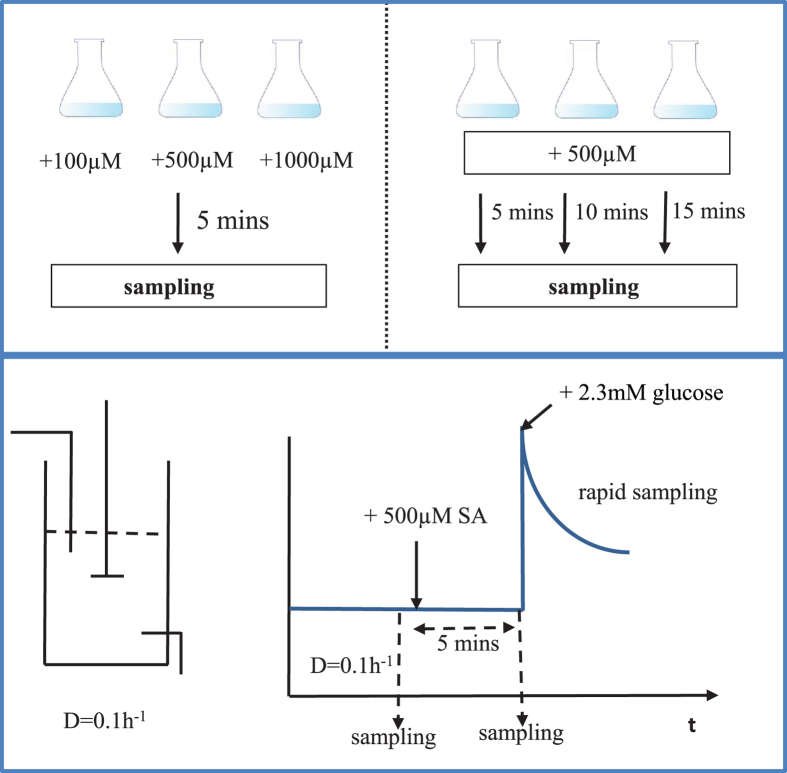
Experiments setup to evaluate the influence. (1) SA addition on intracellular metabolites; (2) SA incubation time on intracellular metabolites; (3) SA uptake and conversion on NADPH production.

Different experiments were performed (Fig. 1) to evaluate the influence of:

1. SA addition on intracellular metabolites;
2. SA incubation time on intracellular metabolites;
3. SA uptake rate and conversion on NADPH production.

The intracellular SA and DHS concentrations were very low in the sensor strain (0.0133 μmol/gDW and below the detection limit, respectively). To increase the intracellular concentrations, SA was added to the culture broth. The addition in batch culture resulted in a 10 fold increased intracellular SA (0.165 μmol/gDW) and measurable DHS (0.0025 μmol/gDW) amounts.

**Figure 2:**
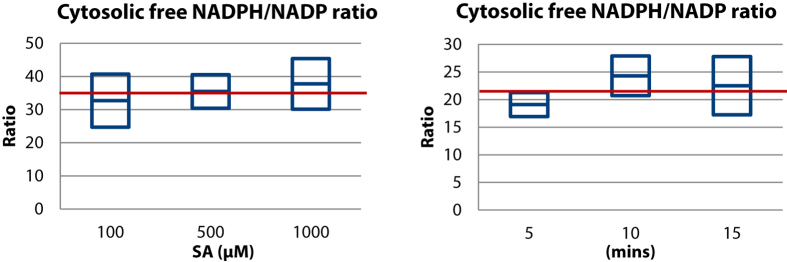
Cytosolic NADPH/NADP ratio in batch experiments. (**A**) Cytosolic NADPH/NADP ratio (based on sensor reaction) during batch growth with different amounts of supplementation, 100, 500 and 1000 μM SA. (**B**) 5, 10 and 15 minutes after addition of 500 μM SA. The error bar represents the standard deviations of the mean of three independent cultivations.

For batch experiments with different amounts of SA addition, the calculated cytosolic free NADPH/NADP ratio was 35.3 ± 2.5, and there was no clear trend for different amounts of SA supplementation in batch condition (Fig. 2A). The cytosolic free NADPH/NADP ratio was very similar although the SA and DHS concentrations were very different. With 100 μM, the intracellular concentrations were still low, thus a supplementation with 500 μM SA was selected for the following experiments.

With different SA incubation time, the average cytosolic free NADPH/NADP ratio was 22.0 ± 2.6 (Fig. 2B). After 10 minutes, the ratio increased slightly and it decreased at 15 minutes. There was no notable difference for the average ratio of 5, 10, 15 minutes, therefore we choose 5 minutes, also in view of the time monitored after the pulse. The ratio was lower compared to the previous batch cultivation, but it has to be noted that a different medium (urea as nitrogen source) was used. Within one medium condition, the results were consistent.

**Figure 3:**
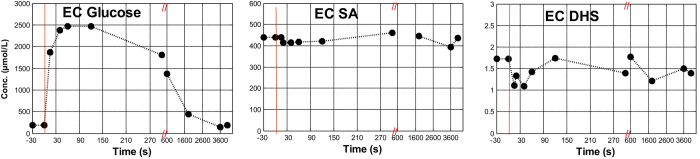
Extracellular glucose, SA and DHS concentrations during the chemostat glucose pulse experiment. Data points before t = 0 represent the state 5 mins after the SA addition, just before the glucose pulse.

Based on these experiments, the supplementation of 500 μM SA and sampling 5 minutes after the addition were chosen for the chemostat experiment. The extracellular addition of SA resulted in a SA uptake rate of 0.005 μmol/(gDW∙s) (Fig. 3), which was calculated as the average rate of 3600 seconds. Assuming that all SA taken up by the cell is dehydrogenated to DHS, an extra NADPH production rate of 0.005 μmol/(gDW∙s) is obtained (~0.29 % of the maximum oxPPP production). It is assumed that this very small putative production has no impact on the metabolic flux distribution, nor the NADPH/NADP ratio. There was no considerable difference of intracellular concentrations of glycolytic and PPP pathway intermediates between the steady state condition and 5 minutes after the addition of SA, except the SA and DSA increased substantially as expected (Supplementary Table S1). Several intermediates in central metabolism were slightly increased, e.g. G6P, F6P, but the difference was not substantial. The results indicate that the intracellular concentration of the sensor reaction metabolites can be boosted by external supplementation without disturbing the metabolic flux distribution.

### Shikimate transport

For *E. coli*, ShiA is identified as high affinity transporter for shikimate which is assumed to function as a proton/shikimate symport^36^. However, the SA transport mechanism in *S. cerevisiae* has not yet been identified. Using the intracellular and extracellular measurement, we could analyse the thermodynamic feasibilities of different putative uptake mechanisms. The equilibrium ratio of SA_in_/SA_out_ is calculated for a range of pH_in_ (6.5–7.0) and pmf (0.15–0.2) for uniport (n = 0), symport (n = 1), and antiport (n = −1) for SA^-^ species and pH_out_ = 5.0. pKa of SA is 4.48.


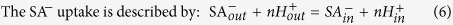


The total acid ratio of SA_in_/SA_out_ at thermodynamic equilibrium can be calculated by:


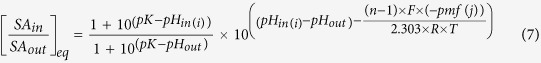


Assuming uniport, an equilibrium ratio of SA_in_/SA_out_ between 0.012 to 0.25 is expected (Supplementary Figure S2). In case of symport, the ratio would be between 24.4 to 78.0. Assuming antiport, the ratio would be between 1 × 10^−5^ to 5 × 10^−4^. In our experiments, an average SA_in_/SA_out_ ratio of 0.3 was found (Supplementary Figure S2). Therefore the transport cannot be antiport, it could be uniport which operates of pseudo equilibrium or symport far from equilibrium. Given the biochemical exchanges and the slow SA uptake rate of 0.005 μmol/(gDW∙s), it is concluded that SA transport occurs as symport, far from equilibrium.

### Steady state cytosolic free and whole cell NADPH/NADP ratio

The NADP biomass specific amount found in different studies are comparable (0.2 ~ 0.5 μmol/gDW), but the discrepancy for NADPH is large, ranging from 0.056 to 1.80 μmol/gDW (Table 1). The whole cell biomass specific amount of NADPH + NADP (0.948 μmol/gDW) was comparable to values reported earlier (Table 1). The steady state whole cell NADPH/NADP ratio was 1.05 ± 0.08, which is similar to the ratio previously reported for *S. cerevisiae* CBS 7336 using enzymatic based measurements^37^; but different from observations in CEN.PK113-7D by enzymatic method (0.29)^17^; and a ratio of 4.8 by LC-MS/MS method^34^. The redox couple NADP(H) is generally considered a challenging target for quantitative analysis^38^. The chemical instability of NADPH in a variety of conditions (especially the sample extraction and process conditions) hinders the accurate determination, while the oxidised form NADP is more stable, even at low pH^39^. The different stabilities are most likely the explanation of the very different reported values of NADPH, while NADP concentrations are comparable.

Based on the metabolite sensor, the glucose-limited steady state cytosolic free NADPH/NADP ratio under aerobic condition (D = 0.1 h^−1^) was 15.6 ± 0.60, which is much higher than the whole cell NADPH/NADP ratio (1.05 ± 0.08). This large difference of NADPH/NADP ratio was also observed in mouse pancreatic islets (26.8 (cytosolic free) vs. 1.2 (whole-cell ratio))^16^. The higher cytosolic free NADPH/NADP ratio is assumed to be due to a large fraction of cytosolic NADP being protein-bound^13^. Recently using another sensor reaction, the *in vivo* free cytosolic NAD/NADH ratio in *S. cerevisiae* was estimated to be higher than 100 under aerobic carbon-limited condition favouring oxidative processes, which was again very different from the whole cell average NAD/NADH ratio of 7.5^15^. We should note that the ratio of redox cytosolic ratios (NADPH/NADP)/(NAD/NADH) is estimated at (15.6/0.01 ≈ 1560), showing a redox Gibbs energy advantage for NADPH of R T ln(1560) = 18.2 kJ/mol. Therefore, these effects cannot be observed by direct measurement of total concentration of NADP(H).

### Cytosolic free and whole cell NADPH/NADP ratio in response to a glucose pulse

**Figure 4:**
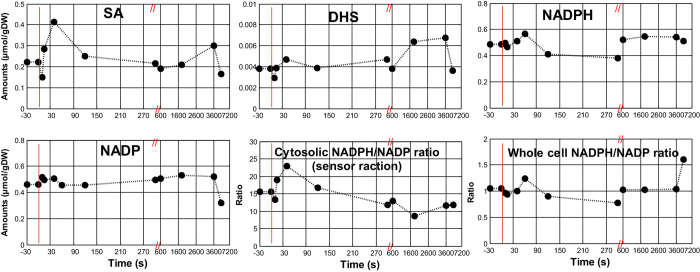
Intracellular SA, DHS, NADPH, NADP amounts, cytosolic and whole cell NADPH/NADP ratio during the pulse experiments. Data points before t = 0 represent the state 5 mins after the SA addition, just before the glucose perturbation.

The extracellular responses of glucose, SA and DHS are shown in Fig. 3. The average uptake rate of glucose in the first 10 minutes was 0.54 μmol/(gDW∙s), and comparable to previous pulse experiments^40^, which is about 1.6 times higher than the steady state (0.33 μmol/(gDW∙s)). There was no considerable changes of extracellular SA in response to the glucose pulse, and the extracellular DHS decreased from 1.72 to 1.10 during the first seconds of the glucose pulse, and increased back to pre-pulse value in two minutes. The intracellular concentration of SA changed about 2 fold (Fig. 4, from 0.22 μmol/gDW to 0.41 μmol/gDW). This shows that the SA transport is not affected by changing intracellular SA level, confirming that SA transport is the irreversible symport mechanism.

After the glucose pulse, the whole cell NADPH increased slightly and then decreased back to the steady state value in 120 seconds. The whole cell NADP remained nearly constant (Fig. 4). The intracellular biomass specific amount of SA increased substantially after the glucose pulse, while there was no substantial change of the DHS level (Fig. 4).

The steady state sensor reaction based cytosolic free NADPH/NADP ratio was 15.6 ± 0.60. During the glucose pulse, the cytosolic ratio decreased first and then increased immediately to 23 (that was found in batch condition) in 1 minute and then decreased back to the pre-pulse level within 3 mins (Fig. 4). The first decrease could due to the delay in oxPPP, because it needs to adjust to the high metabolic flux and the glycolysis is faster. The increase suggests a rapid increase of the flux towards G6PDH and 6PGDH, which could cause the rapid production of NADPH and decrease of free NADP, while NADPH was consumed by the anabolic reactions^41^. Therefore, the cytosolic free NADPH/NADP ratio responded differently to the whole cell NADPH/NADP ratio to a glucose pulse.

### Cytosolic NADPH/NADP related thermodynamics and kinetics

The cytosolic NADPH/NADP ratio is important to elucidate *in vivo* cytosolic pathway kinetics. For G6PDH and 6PGDH of the oxPPP, the overall reaction is:


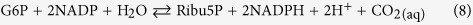


The Gibbs free energy (Δ_r_G′, kJ/mol) was calculated:


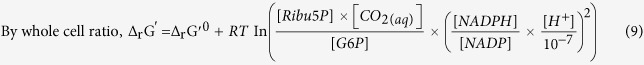


Using the sensor reaction based cytosolic NADPH/NADP ratios:


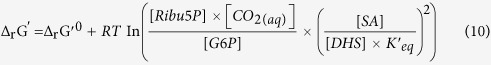


**Figure 5:**
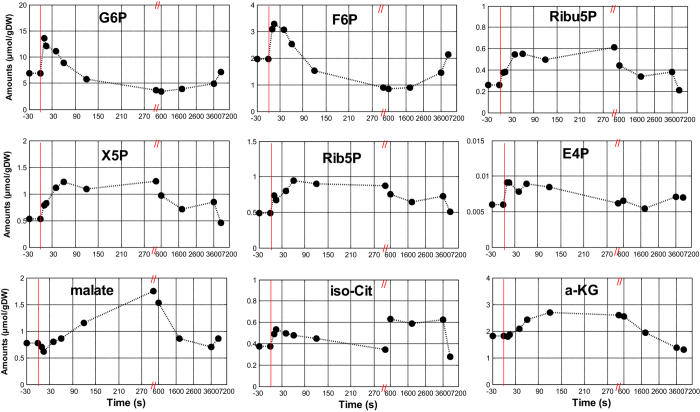
Intracellular metabolite amounts after a glucose pulse. Data points before t = 0 represent the state 5 mins after the SA addition and before the glucose perturbation.

**Figure 6:**
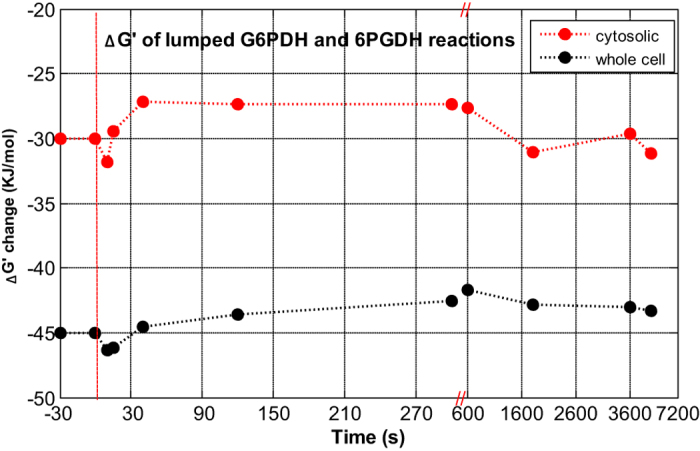
The profile of Gibbs free energy (Δ_r_G′, kJ/mol) for the lumped G6PDH and 6PGDH reactions 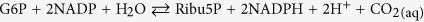
 in the oxPPP by using cytosolic and whole cell NADPH/NADP ratio.

Here, the G6P and Ribu5P were measured (Fig. 5), and note that the Gibbs energy of reaction is not influenced by cytosolic pH because we use the sensor reaction. When *S. cerevisiae* was grown at pH = 5, the concentration of CO_2_ in equilibrium with the concentration of dissolved CO_2_ is in the order of 5% of the dissolved CO_2_ concentration, and therefore the exchange between these two pools was neglected in the calculations^42^,^43^. Assuming that the concentration change of CO_2(aq)_ (estimated of 1 mmol/L using measured off-gas CO_2_ levels) during the pulse is small, the Δ_r_G′ can be estimated by sensor reaction or whole cell NADPH/NADP ratios and metabolite measurements (Fig. 6).

The sensor based estimation of steady state Δ_r_G′ for the oxPPP is −29 kJ/mol, while it is −42 kJ/mol using whole cell ratios. After the pulse, the Δ_r_G′ (by using cytosolic NADPH/NADP ratio) decreased immediately in 10 seconds (about 2 kJ/mol), indicating a higher thermodynamic driving force to allow for a higher flux through the PPP after the pulse. After 10 seconds, Δ_r_G′ increased above the steady state levels, and the thermodynamic driving force is reduced. This is remarkable because it is expected that the PPP flux increases, and this occurrence of reduced driving force suggest that allosteric mechanisms influence the flux. Using whole cell NADPH/NADP ratio measurement, the thermodynamic driving force is mostly due to the concentration changes of Ribu5P and G6P. The response was slower and the change was smaller than using cytosolic NADPH/NADP ratio (Fig. 6). The dynamic changes of other interested metabolites are shown in Fig. 5.

The NADPH producing oxPPP has a very large Δ_r_G′ and is far from the equilibrium, while NADPH consuming reactions are typically close to equilibrium reactions. However, a high driving force is important for the production rate. As mentioned before, one strategy for the microbial production of PDO was to switch NADH-dependent dehydrogenase DhaT to NADPH-dependent dehydrogenase YqhD. The cytosolic free NADH/NAD ratio was estimated to be 0.01 (assuming cytosolic pH 7.0)^15^, while the cytosolic free NADPH/NADP ratio was found to be 15.6 by sensor reaction. The driving force (Δ_r_G′) is then 18.2 kJ/mol larger using NADPH as a cofactor than NADH as a cofactor. This much higher driving force has probably led to a much lower level of the toxic aldehyde 3-HPA, allowing a higher PDO concentrations.

### Model based estimation of fluxes

**Figure 7:**
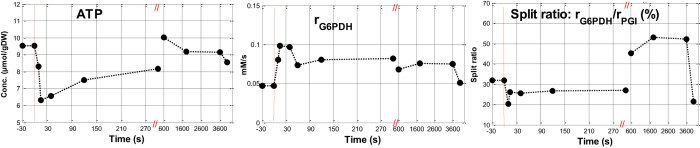
Model based estimation of the intracellular flux of G6PDH and the split ratio of flux into PPP and glycolysis during the pulse experiments. Data points before t = 0 represent the state 5 mins after the SA addition, just before the glucose perturbation.

To obtain an estimation of the dynamic response of the PPP flux, the model of Vaseghi, *et al.*^37^ was used. The steady state and dynamic fluxes into PPP and the flux split ratio into glycolysis and PPP are shown in Fig. 7. The detailed calculation is shown in S1 Appendix.

The steady state glucose uptake rate was 0.194 mM/s. The steady state flux in the PPP was 0.047 mM/s, which was 32% of the flux through the glycolysis (0.194–0.047 = 0.147 mM/s), which is lower than previous reported^37^, but well in the range of 2%–60%^32^,^44^. After the glucose pulse, it increased immediately and achieved a maximum flux of 0.098 mM/s in 15 seconds, and decreased slightly back to the steady state level (Fig. 7).

The glucose uptake rate was high directly after the pulse and decreased in time. Three different phases were distinguished based on the observed NADPH/NADP ratio profile and extracellular profile: 0–60 seconds, 60–600 seconds, 600–1800 seconds. The split ratio into PPP and glycolysis was calculated for the different phases. Both PPP and glycolytic flux increased after the glucose pulse, and the split ratio into PPP decreased from 32% to 20% of the glycolytic flux in 10 seconds and then increased back to the steady-state ratio (Fig. 7). This result is similar but more moderate compared to the observation of immediate decreasing of the split ratio from 56% to 10% after the glucose pulse observed by Vaseghi *et al.*^37^. A similar trend was observed by Frick and Wittmann^45^, here the contribution of the PPP decreased with increasing glucose uptake rate.

The enzyme activity of G6PDH was reported to be inhibited by NADPH/NADP ratio and the ATP level^37^. After the glucose pulse, the ATP concentration decreased (Fig. 7), and the oxPPP reaction rate increased. After 3 minutes, the cytosolic NADPH/NADP ratio dropped below the steady state ratio, which could be due to the decreased G6PDH activity or significantly increased NADPH consumption in biosynthetic reactions.

## Conclusions

The SA-based cytosolic NADPH/NADP sensor reaction was used to determine the cytosolic free NADPH/NADP ratio under different conditions. The sensor reaction reacted rapidly to metabolic perturbations. The cytosolic free NADPH/NADP ratio was 15.6 ± 0.60 in glucose-limited chemostat (D = 0.1 h^−1^) condition, while the measured whole cell NADPH/NADP ratio was more than 10 times lower (1.05 ± 0.08). In response to a glucose pulse, the cytosolic NADPH/NADP ratio increased very rapidly and reached the steady state ratio after about 3 mins, while the whole cell NADPH/NADP ratio didn’t change substantially. The novel cytosolic NADPH/NADP ratio provides new insight on the thermodynamic driving force of NADPH dependent reactions, and will be useful in production strains of reduced products (alcohols, fuels) to identify putative bottlenecks on NADPH supply and problems of thermodynamic driving force in product pathways.

## Materials and Methods

### Strain and plasmid construction

First, a multicopy plasmid for yeast containing the *aroE* gene (encoding shikimate dehydrogenase (SDH)) under expression of the strong promoter *TDH3* was constructed. The *aroE* gene was amplified from *E. coli* DH5α (18258-012, Invitrogen) genomic DNA with PCR using primer pairs SDH forward *pTDH3* overlap (5′ AGTTTCGACGGATTCTAGAACTAGTATGGAAACCTATGCTGTTTTTGGTAATC 3′), SDH reverse *tCYC1* overlap (5′ TAACTAATTACATGACTCGAGTCACGCGGACAATTCCTCCTG 3′) (Sigma-Aldrich) and Phusion^®^ Hot Start II High Fidelity Polymerase (Thermo Scientific, Waltham, MA). The PCR product was purified from 1% agarose (w/v) gel using the Zymoclean™ kit (Zymo Research, Irvine, CA). The plasmid pAG426GPD-ccdB (Addgene, Cambridge, MA, USA) was restricted with SpeI and XhoI (Thermo Scientific), following gel purification of its backbone. The *aroE* cassette was assembled into this backbone using In-Fusion cloning (Clontech, Mountain View, CA, USA). The resulting plasmid pAG426-GPD-SDH was transformed into *E. coli* and confirmed using restriction analysis with SnaBI, SpeI and XhoI (Thermo Scientific). The plasmid was then transformed to *S. cerevisiae* strain CEN.PK113-5D using the lithium acetate protocol^46^, yielding strain CEN.PK-aroE.

### Enzyme activity measurement

The cell extract from the culture samples were prepared by sonication method as described^47^. Protein concentrations in cell extracts were measured with the Lowry method^48^, and bovine serum albumin (BSA) (Sigma-Aldrich, The Netherland) was used as a standard. The activity of shikimate dehydrogenase was assayed in the direction of NADPH production. The assay was conducted in a final volume of 500 μL that contained: 100 mM Tris-HCl, pH 9.0, 4 mM shikimate (Sigma-Aldrich, The Netherland), 2 mM NADP and 5 μL cell extract. The enzyme activity was assayed by continuously monitoring the increase in NADPH absorbance at 340 nm for 30 mins at 25 °C^49^. Additionally, the enzymatic activity of G6PDH was measured according to the protocol of Postma, *et al.*^35^.

### External SA supplementation for the sensor reaction

The intracellular SA and DHS concentrations were too low for accurate measurement in the CEN.PK-aroE strain. Therefore, SA was added to the extracellular space (Fig. 1) . To analyse if there was an influence of SA supplementation on the NADP(H) level, different amounts of SA were added to batch shake flask cultures (NH_4_^+^ as nitrogen source) (100 μM, 500 μM and 1000 μM residual SA). After 5 minutes, the intracellular SA and DHS were measured. To measure if there was an influence of SA incubation time, the supplementation (500 μM) was performed in batch (urea as nitrogen source) and samples were taken after 5, 10 and 15 minutes, respectively. The membrane transport rate and equilibrium was evaluated by a pulse experiment, where a concentrated SA solution (5 mL of 100 mM) was rapidly added to a chemostat culture (1L) using a syringe, resulting in a 500 μM extracellular SA concentration. After 5 minutes, a glucose pulse was added, resulting in a 2.3 mM increase of the residual glucose concentration.

### Media and cultivation

For the working stock, *S. cerevisiae* CEN.PK-aroE strain was cultured aerobically in 500 mL shake flask at 30 °C using 100 mL synthetic medium with 20 g/L glucose as carbon source^50^. After overnight growth, glycerol was added (final concentration 30% (v/v)) and 1 mL aliquots were stored in sterile vials at −80 °C.

Shake flask cultures were performed with synthetic media containing 20 g/L glucose^50^. NH_4_^+^ was used as nitrogen source for the set of batch experiments with/without SA supplementation. Urea was used as nitrogen source for batch experiments with different SA incubation times to avoid decreasing of the extracellular pH.

The glucose-limited chemostat cultivation was performed using a low-salt Verduyn minimal medium^51^ with 7.5 g/L glucose in a 2 L bioreactor (Applikon, The Netherlands) with 1 L working volume. The dilution rate was set to 0.1 h^−1^; pH was kept constant at 5.0 by automatic addition of 2 M KOH as described previously^52^. The temperature was controlled at 30 °C and the head space overpressure was kept at 0.3 bars. The aeration rate was 0.5 vvm and the stirrer speed was 600 rpm. Cultures were assumed to be in steady state after 5 volume changes, as confirmed by constant CO_2_, O_2_ and DO levels. The biomass dry weight concentration was measured as described earlier^34^.

### Glucose pulse experiments in continuous cultivation

To perform the glucose pulse experiment, 5 minutes after the addition of 500 μM SA, a concentrated glucose solution (30 mL of 79 mM glucose) was rapidly added to a chemostat culture using a syringe in glucose-limited chemostat, resulting in a 2.3 mM increase of the residual glucose concentration, which was 0.18 mM. For the continous culture, the steady samples were measured in triplicate by taking three independent samples from the same cultivation. During the dynamic glucose pulse experiment, only single samples could be taken and this experiment has only been performed once (very labor intensive). Please take into account that timeseries measurements contain a certain level of redundancy because of the dense sampling in time.

### Intracellular metabolites

The sampling, quenching, extraction and analysis of the intracellular metabolites was similar to the methods described earlier in Wahl, *et al.*^53^ using a rapid sampling device. Briefly, 1.2 mL broth was withdrawn from the bioreactor and immediately quenched in 6 mL cold (−40 °C) 100% methanol, followed by vortexing and weighing. For shake flask cultures, samples were taken directly from the flask by pipetting and immediate quenching in 6 mL cold (−40 °C) 100% methanol. Clearly, this latter sampling was slower compared to the rapid sampling device, nevertheless batch and chemostat are different conditions and we expect representative results with this approach because the samples contain excess substrate and oxygen limitation is not reached during the transfer of about 3 seconds. 120 μL of ^13^C cell extract were added to the tube as an internal standard^54^. The intracellular metabolites were extracted and analysed by GC-MS^55^ or LC-MS/MS^56^,^57^, including whole cell NADPH and NADP concentrations^58^. The SA and DHS were analysed by GC-MS/MS, and the collision energy (CE) for SA is 35 and for DHS is 5 eV. The MS/MS fragments for SA is 372.1/73.1(^12^C) and 379.1/73.1(^13^C, internal standard), and the MS/MS fragments for DHA is 417.1/386.1 (^12^C) and 424.1/393.1(^13^C). Amounts were converted to intracellular concentrations by using a cellular volume of 1.7 mL/gDW^59^. Please note that in this work, we focus on the NADP/NADPH ratio. Therefore only the concentration ratio is the determining factor. Even if the volume assumption was severely violated, the amount of SA/DHS will be correct as all cytosolic concentrations are affected by the conversion (from the raw data μmol/gDW to cytosolic concentration).

### Extracellular metabolites

Samples for extracellular metabolite analysis were taken using the cold steel beads method as described earlier^60^. The concentration of glucose was determined by HPLC, GC-MS or enzymatically as described elsewhere^34^. The extracellular concentrations of SA and DHS were determined by GC-MS/MS. Here, an aliquot of 100 μL sample was mixed with 20 μL of^13^C cell extract and further processed following the method described in Wahl, *et al.*^53^.

## Additional Information

**How to cite this article**: Zhang, J. *et al.* Determination of the Cytosolic NADPH/NADP Ratio in *Saccharomyces cerevisiae* using Shikimate Dehydrogenase as Sensor Reaction. *Sci. Rep.* **5**, 12846; doi: 10.1038/srep12846 (2015).

## Supplementary Material

Supporting Information

## Figures and Tables

**Table 1:** Comparison of NADP(H) results.

Strain	Vaseghi, *et al*.^37^	Moreira dos Santos, *et al*.^17^	Suarez-Mendez, *et al*.^34^	This work
	CBS 7336	CEN.PK113-7D**	CEN.PK113-7D	CEN.PK-aroE
Cultivation condition	Glucose-limited, aerobic, D = 0.1 h^−1^			
Extraction method	alkaline and acidic solvent	alkaline and acidic solvent	boiling ethanol	boiling ethanol
Analysis	enzymatic assay	enzymatic assay	LC-MS/MS	LC-MS/MS
NADPH (μmol/gDW)	~0.289*	0.056	1.80	0.486
NADP (μmol/gDW)	~0.281*	0.19	0.37	0.462
NADPH + NADP (μmol/gDW)	0.55	0.246	2.17	0.948
NADPH/NADP ratio	1.03	0.29	4.86	1.05

^*^Amounts were converted to μmol/gDW by using a cellular volume of 1.7 mL/gDW.

^**^The cultivation presented persistent metabolic oscillations.
