# Supplementary material for: Determination of the Cytosolic NADPH/NADP Ratio in Saccharomyces cerevisiae using Shikimate Dehydrogenase as Sensor Reaction
Source: Sci Rep. 2015 Aug 5;5:12846. doi: 10.1038/srep12846 (PMC4525286; doi:10.1038/srep12846)
Supplement: Supporting Information [file srep12846-s1.pdf]

Determination of Cytosolic NADPH/NADP Ratio in *Saccharomyces cerevisiae* using Shikimate Dehydrogenase as Sensor Reaction

Jinrui Zhang[1,*], Angela ten Pierick[1], Harmen M. van Rossum[1], Reza Maleki Seifar[1], Cor Ras[1], Jean-Marc Daran[1], Joseph J. Heijnen[1], and S. Aljoscha Wahl[1,*]

Supplementary Information

Supplementary Table S-1. The steady state intracellular metabolite concentrations of aerobic glucose-limited chemostat culture at D=0.1 h^{-1} for the reference and CEN.PK-aroE strains. For CEN.PK-aroE, two states are included, steady state and 5 minutes after adding the shikimate to the steady state. Data were average of three replicate samples ± standard deviation.

Intracellular concentration (µmol/gDW)	CEN.PK113-7D* Steady state	CEN.PK-aroE Steady state	CEN.PK-aroE 5 minutes after adding SA to steady state	P value**	P value***
G6P	5.42 ± 0.26	5.62 ± 0.28	6.08 ± 0.50	0.58	0.26
F6P	1.50 ± 0.08	1.60 ± 0.05	1.75 ± 0.12	0.25	0.15
DHAP	0.54 ± 0.03	0.69 ± 0.07	0.79 ± 0.03	**0.03**[#]	0.15
GAP	0.032 ± 0.017	0.034 ± 0.004	0.037 ± 0.014	0.96	0.66
3PG	4.99 ± 0.29	5.29 ± 0.15	5.20 ± 0.05	0.350	0.49
Ribu5P	0.28 ± 0.01	0.22 ± 0.01	0.23 ± 0.02	0.07	0.56
X5P	0.61± 0.02	0.46 ± 0.03	0.49 ± 0.01	0.10	0.35
Fumarate	0.67 ± 0.02	0.76 ± 0.07	0.71 ± 0.02	0.35	0.44
Malate	3.01 ± 0.08	3.05 ± 0.12	3.07 ± 0.11	0.72	0.84
Shikimate	n.a	0.0133 ± 0.001	0.165 ± 0.021	n.a	**0.007**[#]

Dehydroshikimate	n.a	Too low	0.0025 ± 0.0005	n.a	n.a

*data from Suarez-Mendez, et al. [34]. n.a, not analysed.

** P value between steady state values of wildtype and CEN.PK-aroE strain.

*** P value between steady-state and 5 minutes after adding SA of CEN.PK-aroE strain.

[#] Significant difference.

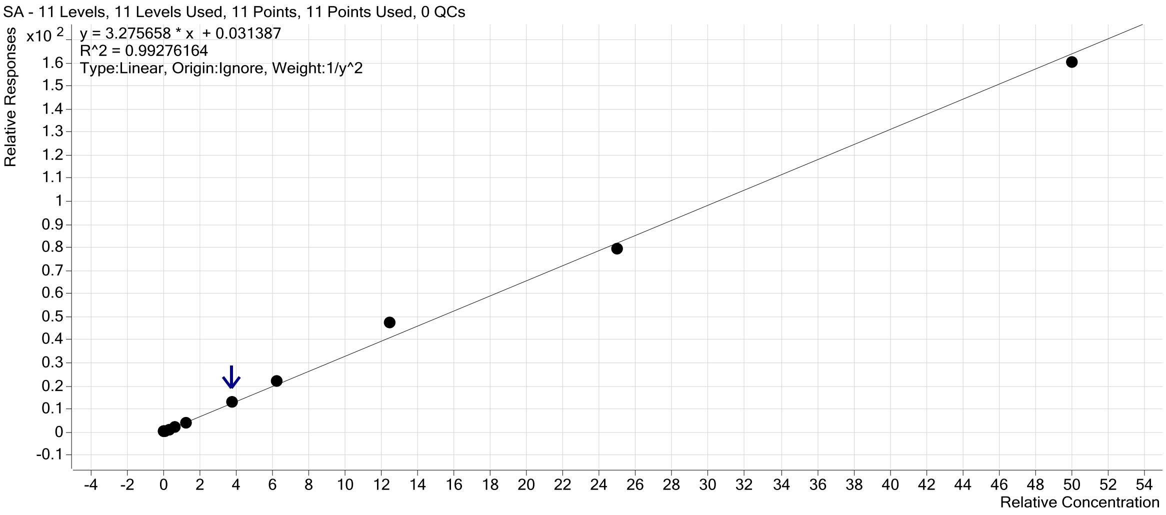

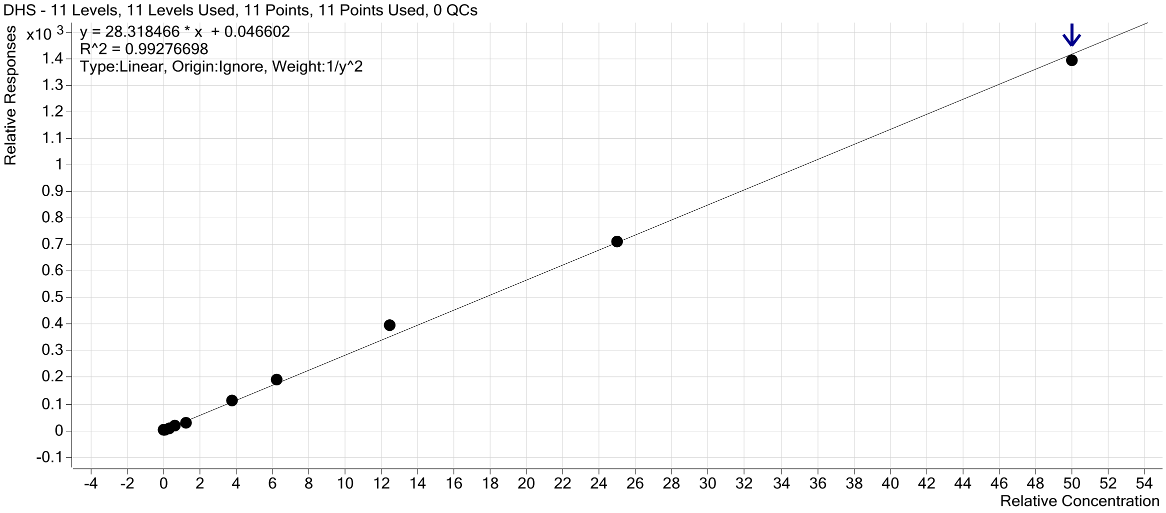

Supplementary Figure S1. The calibration lines of the standards of SA (top) and DHS (below). Both are linear in the range of 0.0313 µM to 50µM.

More details on the GC-MS/MS method developed by our group is submitted in a different context to Biotechnology Journal: Impact of natural isotopes in metabolomics and ^{13}C fluxomics:

an update for MS/MS. S. Niedenführ, A. ten Pierick, P. van Dam, C. Suarez-Mendez, K. Nöh, S.A. Wahl. Biotechnology Journal (under review).

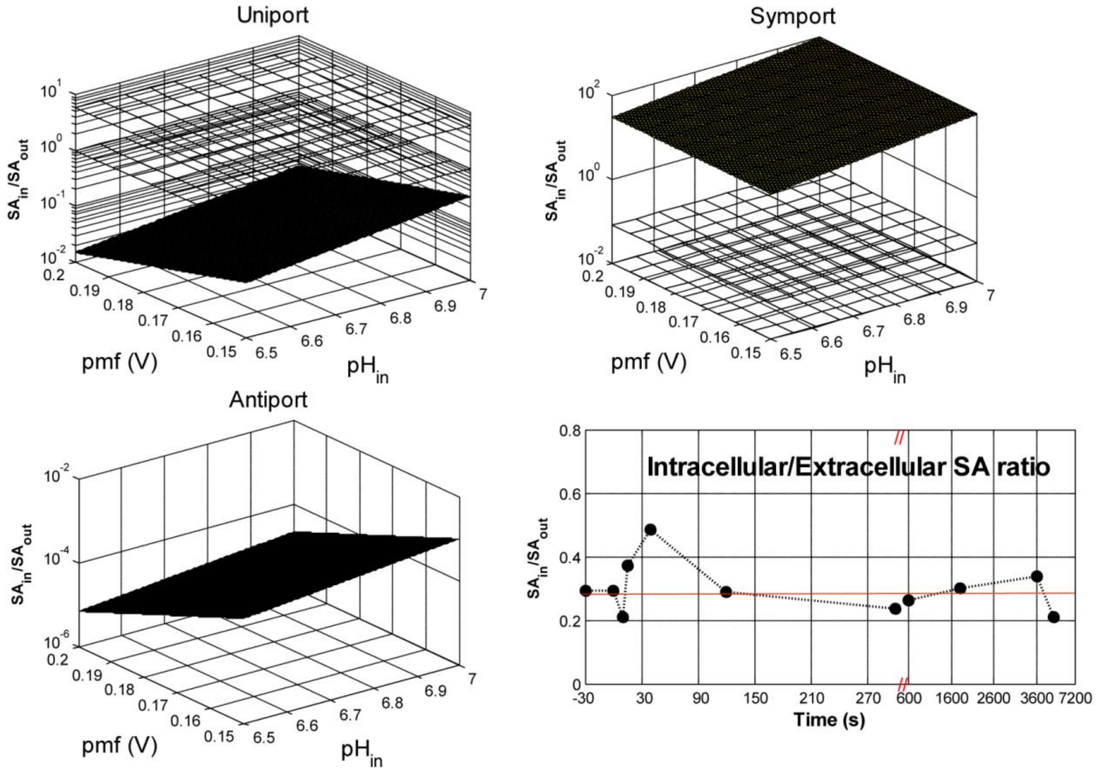

Supplementary Figure S2. SA_{in}/SA_{out} transport. (A) The equilibrium ratio of SA_{in}/SA_{out} for a range of pHin (6.5-7.0) and pmf (0.15-0.2). Assuming uniport, an equilibrium ratio between 0.012 to 0.25 is expected. In case of symport, the ratio would be between 24.4 to 78.0. Assuming antiport, the ratio would be between $1*10^{-5}$ to $5*10^{-4}$. (B) Experimental SA_{in}/SA_{out} ratio (average 0.3). Therefore, the SA transport in *S. cerevisiae* most likely functions as a uniport. It could also be a proton/shikimate symport and it is far from equilibrium.
